# Serum adipokine profiles in patients with microscopic polyangiitis and granulomatosis with polyangiitis: An exploratory analysis

**DOI:** 10.1371/journal.pone.0254226

**Published:** 2021-07-09

**Authors:** Sung Soo Ahn, Taejun Yoon, Jason Jungsik Song, Yong-Beom Park, Sang-Won Lee

**Affiliations:** 1 Department of Internal Medicine, Yongin Severance Hospital, Yonsei University College of Medicine, Yongin, Republic of Korea; 2 Department of Medical Science, BK21 Plus Project, Yonsei University College of Medicine, Seoul, Republic of Korea; 3 Division of Rheumatology, Department of Internal Medicine, Yonsei University College of Medicine, Seoul, Republic of Korea; 4 Institute for Immunology and Immunological Diseases, Yonsei University College of Medicine, Seoul, Republic of Korea; Soroka University Medical Center, ISRAEL

## Abstract

**Objectives:**

Previous studies have shown that adipokines may serve as potential biomarkers reflecting disease activity in various autoimmune diseases. Here, we investigated the relationship between four adipokines and clinical/laboratory findings in patients with microscopic polyangiitis (MPA) and granulomatosis with polyangiitis (GPA).

**Methods:**

Sera from 63 patients with MPA and GPA who were registered in a prospective cohort were used to detect serum levels of adiponectin, chemerin, resistin, and vaspin using commercial enzyme-linked immunosorbent assay kits. Associations between adipokines and clinical and laboratory data was assessed using Pearson’s correlation analysis.

**Results:**

The median age was 65.0 years, 24 patients were male, and 42 patients were diagnosed with MPA. The median levels of adiponectin, chemerin, resistin, and vaspin in patient sera were 13.9 ng/mL, 9.2 ng/mL, 23.7 ng/mL, and 0.1 ng/mL, respectively. A significant correlation between chemerin level and five-factor score (FFS) was found (r = 0.320, p = 0.011), and resistin was correlated with both Birmingham vasculitis activity score and FFS (r = 0.256, p = 0.043 and r = 0.320, p = 0.011). Regarding laboratory data, adiponectin level was associated with creatinine, and chemerin level was associated with creatinine, albumin, and erythrocyte sedimentation rate (ESR). On the other hand, resistin was found to be associated with white blood cell count, creatinine, ESR, and C-reactive protein. Age did not have a significant impact on the levels of adipokines.

**Conclusions:**

The expression of adipokines in the sera of patients with MPA and GPA differs depending on clinical and laboratory features, and serum resistin may be suggested as a potential biomarker reflecting disease activity.

## 1. Introduction

Anti-neutrophil cytoplasmic antibody (ANCA)-associated vasculitis (AAV) is a rare systemic autoimmune disease characterized by the occurrence of necrotic inflammation within the small vasculatures [1]. AAV is categorized into three groups; microscopic polyangiitis (MPA), granulomatosis with polyangiitis (GPA), and eosinophilic granulomatosis with polyangiitis (EGPA), according to the clinical and histopathological findings and the type of ANCAs detected [2]. Even though overlapping pathogenesis is present among the three types of AAV, MPA and GPA are considered to be more closely linked than EGPA. This is because the clinical features of MPA and GPA are similar, and distinct allergic features and infiltration of eosinophils into the affected tissues are present in EGPA [3].

Epidemiologic studies indicate that AAV usually occurs in the ages of 60s and 70s, and its clinical course is often unfavorable because of its aggressive nature [4]. Therefore, the treatment strategy for AAV is mainly to control active disease and maintain remission. Therefore, adequate assessment of disease status is essential and several instruments, such as the Birmingham vasculitis activity score (BVAS), BVAS/Wegener’s granulomatosis, physician global assessment, disease extent index, five-factor score (FFS), and vasculitis activity index, have been developed to assess the current disease status in AAV [5–7]. Among these indices, BVAS and its revisions are currently the standard to measure disease activity in AAV [8]. However, the major weakness of BVAS is that specific training is required to assess BVAS accurately, and the calculations take a long time. Consequently, the need to develop biomarkers to assess AAV disease activity still exists.

The adipose tissue is traditionally regarded as a metabolic organ that is responsible for storing energy; however, recent evidences indicate that adipose tissue plays an essential role in homeostasis by linking the body and the immune system via the secretion of cytokines called adipokines [9]. Subsequent to the discovery of leptin in 1994 [10], it has been determined that a variety of adipokines, such as the adiponectin, chemerin, resistin, visfatin, and vaspin, circulate in the peripheral blood and exert either pro-inflammatory or anti-inflammatory immune effects [11]. In line with this, several studies have demonstrated that adipokines are potential biomarkers in various autoimmune diseases, such as rheumatoid arthritis (RA), systemic lupus erythematosus (SLE), and psoriasis [12–14]. However, the association between adipokines and disease activity in patients with AAV, especially in those with MPA and GPA, is not entirely understood. Therefore, in the present study, we aimed to evaluate the relationship between four adipokines and clinical and laboratory findings in the sera of patients with MPA and GPA.

## 2. Materials and methods

### 2.1. Patient inclusion and clinical data acquisition

This was a pilot study that included a total of 63 patients with MPA and GPA, who were enrolled in the Severance Hospital ANCA-associated VasculitidEs (SHAVE) cohort between November 2016 and June 2019. Patients in the SHAVE cohort with AAV (MPA, GPA, and EGPA) were enrolled and followed up regularly for three to six months. On every visit, laboratory and imaging tests and clinical and laboratory data assessment were performed. The patients were classified as MPA and GPA based on the 2007 European Medicine Agency algorithm and the modifications of the 2012 Chapel Hill Consensus Conference definitions [3, 15].

For clinical data, demographic characteristics, including age, sex, weight, body mass index (BMI), diagnosis, disease duration, BVAS (version 3), FFS (2009), and the Korean version of short form-36, were assessed; the clinical features were recorded based on the nine subcategories of the BVAS [5, 16]. Medications that were administered were tallied at the date of clinical data acquisition. The serum samples were collected from the whole blood of each patient at the date of clinical assessment. The samples were stored at -70°C after centrifugation until further analysis and written informed consent was obtained from the patients at the time of blood sampling. This study was approved by the Severance Hospital’s Institutional Review Board, and conducted according to the principles set forth in the Declaration of Helsinki (4-2016-0901).

### 2.2. Laboratory data collection and analysis of adipokines, interleukin (IL)-6, and tumor necrosis factor (TNF)-α levels

On the day of clinical data assessment, the following inflammatory marker data were collected: white blood cell (WBC) count, neutrophil count, platelet count, creatinine level, serum albumin level, erythrocyte sedimentation rate (ESR), and C-reactive protein (CRP) level. In addition, fasting levels of glucose, total cholesterol, triglyceride, high-density lipoprotein (HDL), and low-density lipoprotein (LDL), as well as the ANCA status, were obtained. We estimated the serum levels of adipokines, IL-6, and TNF-α using stored patient sera. Analysis of serum adipokine levels was conducted using multiplex cytokine assays (LXSAHM, R&D Systems, MN, USA), and IL-6 (D6050, R&D Systems, MN, USA) and TNF-α (DTA00D, R&D Systems, MN, USA) levels were measured using enzyme-linked immunosorbent assay (ELISA) kits according to the manufacturer’s instructions. Serum samples from 40 healthy subjects was also used to analyze the level of adipokines in controls.

### 2.3. Statistical analyses

Statistical analyses were performed using the MedCalc statistical software version 19 (MedCalc Software, Ostend, Belgium). The continuous and categorical variables are presented as median (interquartile range [IQR]) and percentages. The differences between continuous variables were determined using Kruskal-Wallis test or the Mann-Whitney U test. High BVAS and FFS were defined as BVAS ≥ 12 and FFS ≥ 2, as previously described [17, 18], and the correlations between continuous variables were determined using Pearson’s correlation analysis. The cut-off values of adipokine levels to differentiate high BVAS and FFS were calculated using the area under the receiver-operating-characteristic curve and the Youden index. For all statistical analyses, a two-tailed p-value of < 0.05 was regarded statistically significant.

## 3. Results

### 3.1. Basal characteristics of patients

The basal characteristics of patients are shown in Table 1. The median age and BMI of patients was 65.0 years and 22.4, and 24 (38.1%) patients were male. Forty-two (66.7%) patients were diagnosed with MPA, and the median disease duration was 1.0 months. The median BVAS and FFS of the patients were 7.0 and 2.0, respectively. Regarding the clinical features, pulmonary (60.3%) and renal (58.7%) manifestations were the most common. Glucocorticoid treatment was the most common (71.4%) among patients, followed by azathioprine treatment (22.2%) and cyclophosphamide treatment (19.0%); however, 18 (28.6%) patients were not treated with any immunosuppressive agent.

**Table 1:** Clinical and laboratory characteristics of patients with MPA and GPA (n = 63).

Variables	p-value
**Clinical data**	
**Demographic characteristics**	
Age (years)	65.0 (53.3–73.0)
Weight, kg	55.6 (50.0–65.8)
BMI, kg/m^2^	22.4 (20.2–24.9)
Male sex, n (%)	24 (38.1)
MPA diagnosis, n (%)	42 (66.7)
Disease duration (months)	1.0 (0.0–19.8)
BVAS	7.0 (4.3–16.0)
FFS	2.0 (1.0–2.0)
SF-36 PCS	47.5 (31.4–66.6)
SF-36 MCS	55.6 (39.3–71.2)
**Clinical features, n (%)**	
General	20 (31.7)
Cutaneous	7 (11.1)
Mucous membranes/eyes	5 (7.9)
Ear Nose Throat	20 (31.7)
Pulmonary	38 (60.3)
Cardiovascular	2 (3.2)
Gastrointestinal	0 (0.0)
Renal	37 (58.7)
Nervous	11 (17.5)
**Current medications, n (%)**	
Glucocorticoids	45 (71.4)
Cyclophosphamide	12 (19.0)
Azathioprine	14 (22.2)
Rituximab	2 (3.2)
Methotrexate	2 (3.2)
Tacrolimus	2 (3.2)
Mycophenolate mofetil	1 (1.6)
None	18 (28.6)
**Laboratory data**	
**Inflammatory markers**	
White blood cell count (/mm^3^)	7.8 (6.3–11.0)
Neutrophil count (/mm^3^)	5.9 (4.3–10.0)
Platelet (× 1,000/mm^3^)	252.0 (210.3–351.0)
Creatinine (mg/dL)	1.0 (0.7–3.0)
Serum albumin (g/dL)	3.6 (3.1–4.0)
ESR (mm/h)	44.0 (23.5–69.0)
CRP (mg/L)	3.0 (0.8–19.0)
TNF-α positivity	8 (12.7)
IL-6 positivity	51 (81.0)
**Glucose and lipid profiles**	
Glucose level (mg/dL)	104.0 (86.3–127.8)
Total cholesterol (mg/dL)	193.5 (164.3–220.9)
Triglyceride (mg/dL)	112.0 (83.3–158.3)
HDL cholesterol (mg/dL)	57.0 (40.0–68.5)
LDL cholesterol (mg/dL)	107.0 (89.5–129.5)
**ANCA status (N, (%))**	
MPO-ANCA (or P-ANCA) positivity	42 (66.7)
PR3-ANCA (or C-ANCA) positivity	6 (9.5)
ANCA negativity	15 (23.8)

Values are expressed as median (interquartile ranges) or in number (percentage).

MPA: Microscopic polyangiitis, GPA: Granulomatosis with polyangiitis, BMI: Body mass index, BVAS: Birmingham vasculitis activity score, FFS: Five factor score, SF: Short form, PCS: Physical component summary, MCS: Mental component summary, ESR: Erythrocyte sedimentation rate, CRP: C-reactive protein, TNF: Tumor necrosis factor, IL: Interleukin, HDL: High density lipoprotein, LDL: Low density lipoprotein, ANCA: Antineutrophil cytoplasmic antibody, MPO: Myeloperoxidase, P: Perinuclear, PR3: Proteinase 3, C; Cytoplasmic.

**Fig 1:**
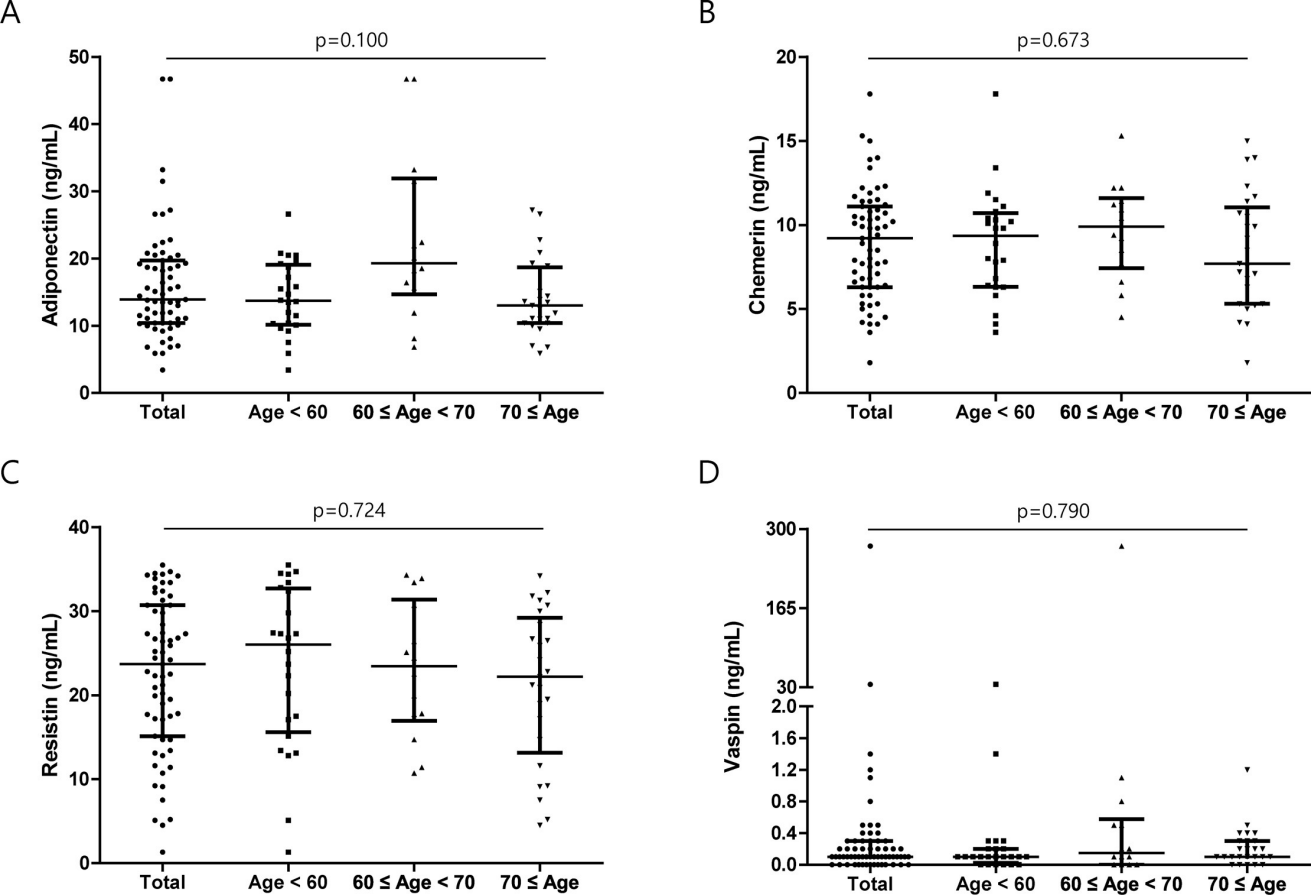
Comparison of adipokine serum levels between age groups. Serum levels of (A) adiponectin, (B) chemerin, (C) resistin, and (D) vaspin were compared by dividing the patients into three groups of age < 60, 60 ≤ age < 70, 70 ≤ age. Error bars indicate median and interquartile ranges.

**Fig 2:**
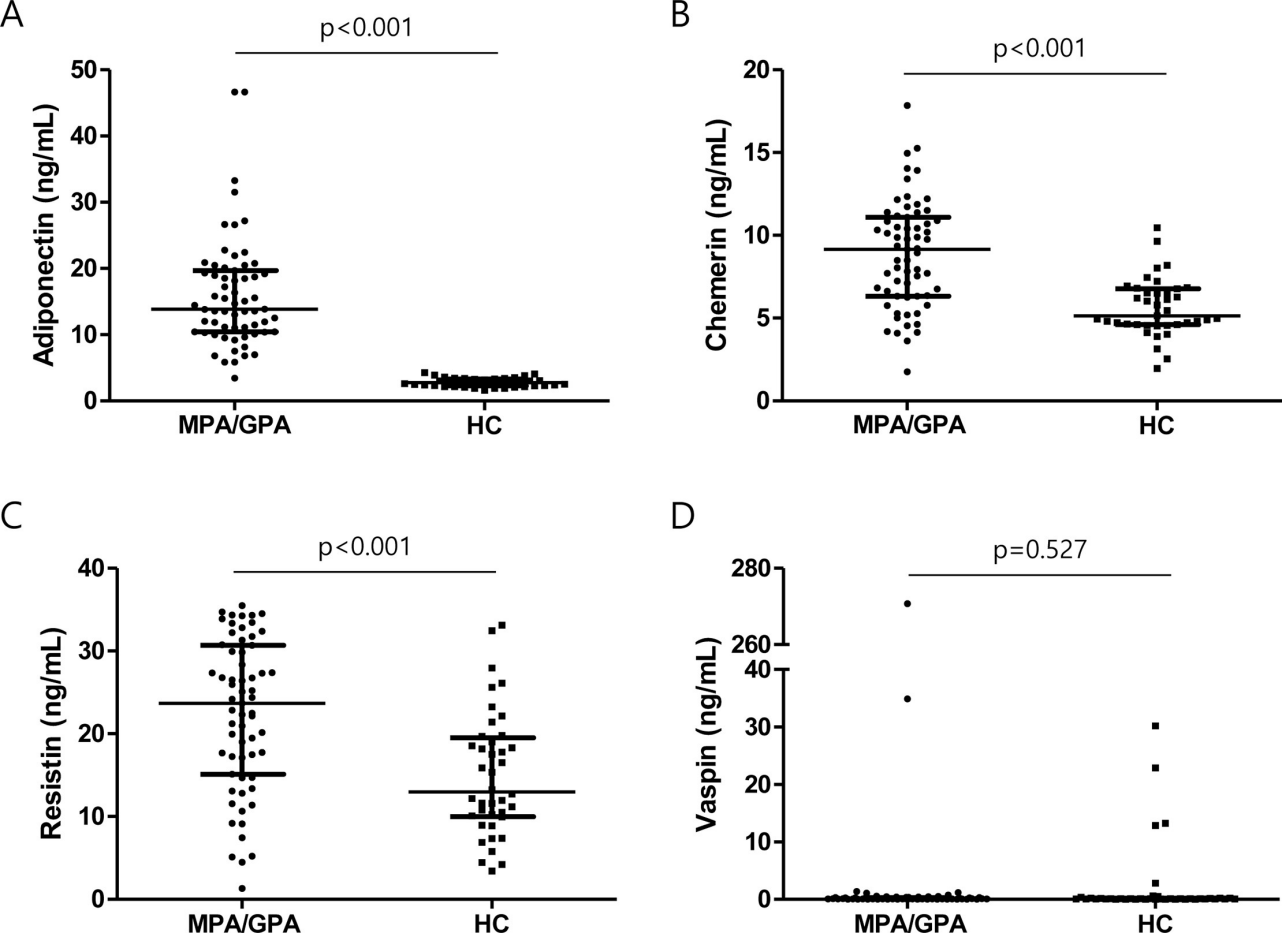
Serum adipokine levels in patients with MPA and GPA compared to healthy controls. When compared to healthy controls, serum adipokine levels of (A) adiponectin, (B) chemerin, and (C) resistin were significantly higher in patients with MPA and GPA, whereas no difference was found in (D) vaspin levels. Error bars indicate median and interquartile ranges. MPA: Microscopic polyangiitis, GPA: Granulomatosis with polyangiitis, HC: Healthy control.

The median levels of WBC count, ESR, and CRP were 7.8/mm^3^, 44.0 mm/h, and 3.0 mg/L, respectively. Eight (12.7) and 51 (81.0%) patients were TNF-α and IL-6 positive, respectively. More than 60% of patients were positive for myeloperoxidase (MPO) or perinuclear ANCA, whereas proteinase 3 (PR3) or cytoplasmic ANCA was found in less than 10% patients (Table 1). The median levels of adiponectin, chemerin, resistin, and vaspin were 13.9 ng/mL, 9.2 ng/mL, 23.7 ng/mL, and 0.1 ng/mL, respectively (Fig 1). Among the level of adipokines, serum adiponectin, chemerin, and resistin was significantly higher in patients with MPA and GPA compared to healthy controls (HCs) (all p<0.001) (Fig 2).

### 3.2. Correlations between adipokines, clinical/laboratory data, and glucose and lipid profiles

The serum chemerin level was correlated with FFS (r = 0.320, p = 0.011), whereas the serum resistin level was correlated with both BVAS and FFS (r = 0.256, p = 0.043 and r = 0.320, p = 0.011). Among the adipokine levels, only serum resistin levels were significantly correlated with BVAS (Table 2). The receiver-operating-characteristic analysis showed that serum resistin levels could predict high BVAS (area under the ROC curve 0.660, 95% confidence interval 0.530–0.774, p = 0.021) but not high FFS (S1A and S1B Fig). Furthermore, serum level of adiponectin was correlated with creatinine and chemerin level was correlated with creatinine, albumin, and ESR. On the other hand, the resistin level was found to be correlated with WBC count, creatinine, ESR, and CRP levels (Table 2).

**Table 2:** Associations between adipokines and clinical and laboratory data.

	Adiponectin	Chemerin	Resistin	Vaspin
Weight	-0.121 (0.345)	-0.068 (0.595)	0.033 (0.797)	-0.179 (0.162)
BMI	-0.106 (0.409)	-0.060 (0.641)	-0.084 (0.515)	-0.233 (0.066)
BVAS	0.045 (0.727)	0.127 (0.321)	0.256 (0.043)	-0.061 (0.637)
FFS	0.130 (0.309)	0.320 (0.011)	0.320 (0.011)	0.195 (0.126)
SF-36 PCS	-0.111 (0.388)	-0.136 (0.287)	-0.124 (0.332)	0.001 (0.993)
SF-36 MCS	-0.165 (0.197)	-0.040 (0.756)	-0.040 (0.756)	0.116 (0.366)
White blood cell count	-0.081 (0.529)	-0.103 (0.421)	0.277 (0.028)	-0.028 (0.830)
Neutrophil count	0.017 (0.897)	0.097 (0.449)	0.136 (0.290)	-0.054 (0.676)
Platelet	-0.074 (0.566)	0.135 (0.290)	0.188 (0.141)	-0.087 (0.496)
Creatinine	0.387 (0.002)	0.319 (0.011)	0.457 (<0.001)	0.162 (0.204)
Albumin	0.031 (0.807)	-0.285 (0.024)	-0.241 (0.057)	0.047 (0.716)
ESR	0.009 (0.942)	0.396 (0.001)	0.327 (0.009)	0.041 (0.752)
CRP	-0.085 (0.509)	0.140 (0.274)	0.268 (0.033)	0.114 (0.372)

Numbers indicate correlation coefficients and p-values are presented in parentheses.

BVAS: Birmingham vasculitis activity score, FFS: Five factor score, SF: Short form, PCS: Physical component summary, MCS: Mental component summary, ESR: Erythrocyte sedimentation rate, CRP: C-reactive protein.

Given that adipokines are closely linked to the changes of metabolism, the relationship between adipokines with glucose and lipid profiles were thus analyzed [19]. Within laboratory data comprising glucose and lipid profiles, only HDL cholesterol was found to be significantly correlated with adiponectin level (r = 0.417, p<0.001) (Table 3).

**Table 3:** Correlations between adipokines, glucose, and lipid profiles.

	Adiponectin	Chemerin	Resistin	Vaspin
Glucose level	-0.166 (0.193)	-0.036 (0.780)	0.134 (0.295)	-0.096 (0.455)
Total cholesterol	0.213 (0.094)	-0.158 (0.215)	-0.136 (0.289)	0.100 (0.436)
Triglyceride	-0.212 (0.095)	-0.154 (0.228)	0.149 (0.245)	-0.020 (0.875)
HDL cholesterol	0.417 (<0.001)	-0.201 (0.115)	-0.204 (0.108)	0.044 (0.729)
LDL cholesterol	0.053 (0.683)	-0.110 (0.391)	-0.135 (0.293)	0.065 (0.611)

Numbers indicate correlation coefficients and p-values are presented in parentheses.

HDL: High density lipoprotein, LDL: Low density lipoprotein.

### 3.3. Correlations between serum adipokine levels and age, as well as TNF-α and IL-6 status

Since age influences the levels of adipokines [20], we divided our patients into three groups, younger than 60 years (age < 60), between the ages of 60 and 70 (60 ≤ age <70), and older than 70 years (70 ≤ age), and compared the serum levels of adipokines. However, no correlation was found between serum adipokine levels and age (Fig 1A–1D).

**Fig 3:**
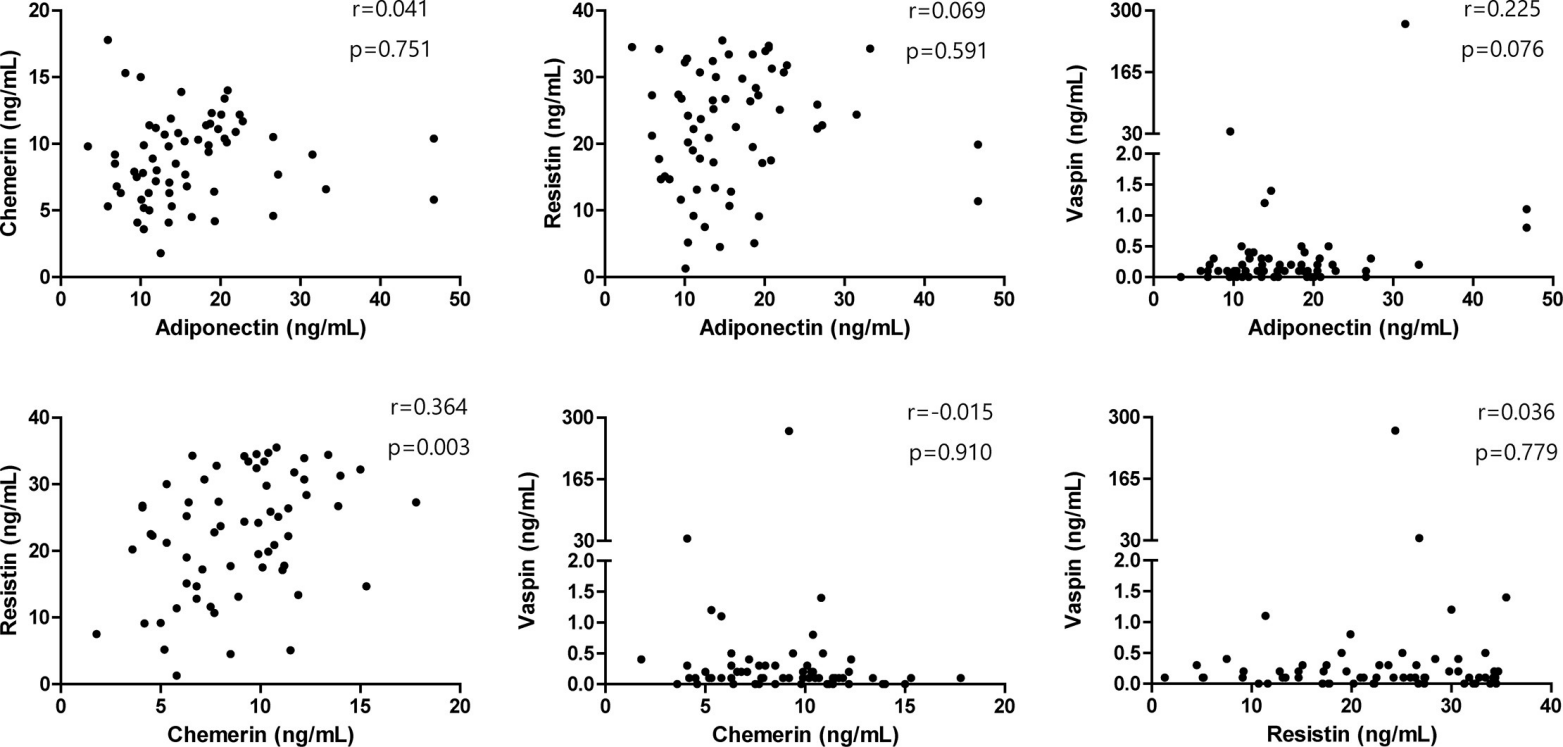
Assessment of the correlations between adipokine levels. The correlations between serum adipokine levels were assessed.

**Fig 4:**
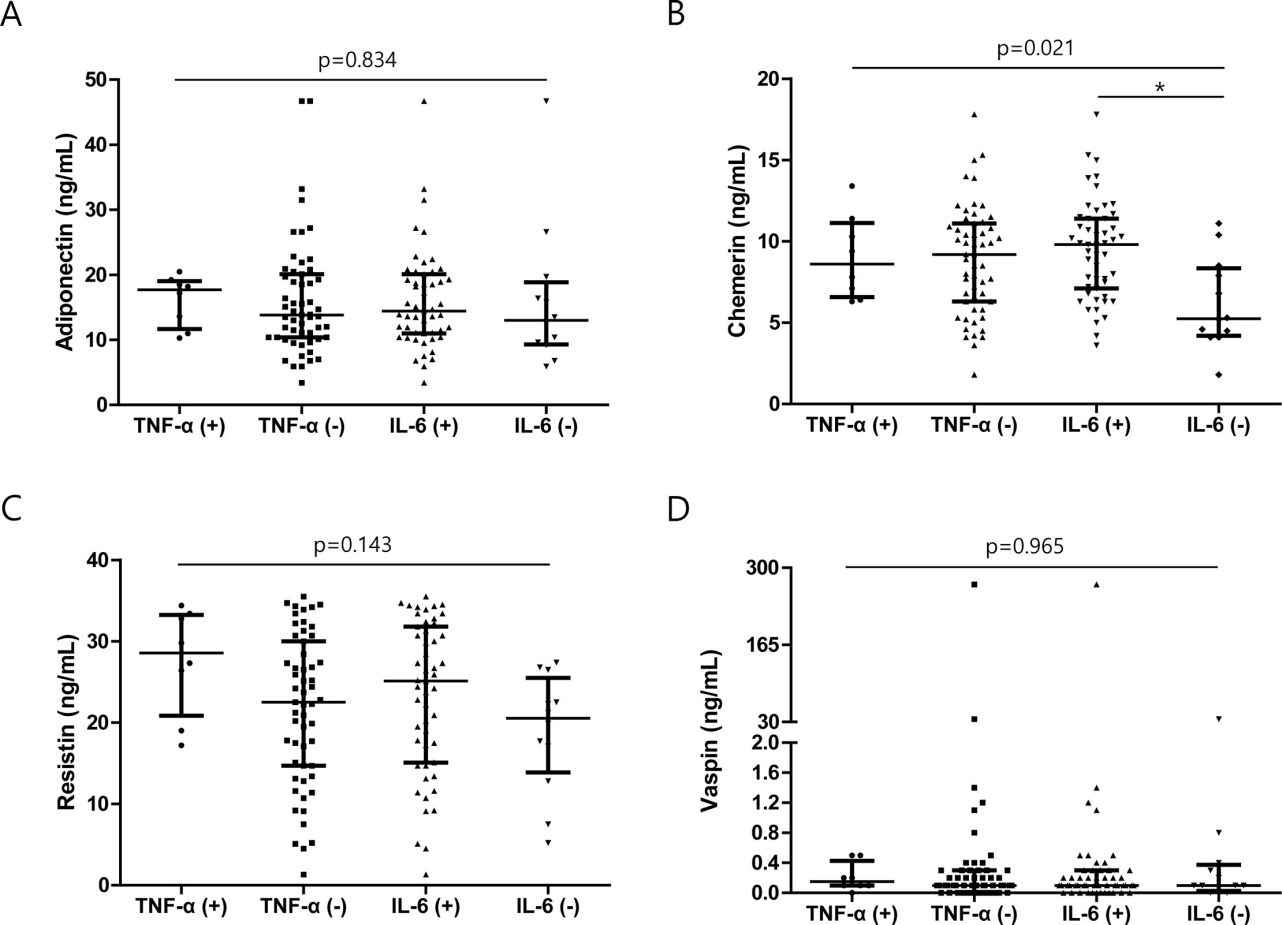
Association of serum adipokine levels with TNF-α and IL-6 positivity or negativity. Serum levels of (A) adiponectin, (B) chemerin, (C) resistin, and (D) vaspin were compared between TNF-α and IL-6 positive and negative cases. Error bars indicate median and interquartile ranges. TNF: Tumor necrosis factor, IL: Interleukin.

Besides, as it is being regarded that adipokines form a complex networking system to influence the immune system [21], the association between serum levels of different adipokines was investigated. Adiponectin level tended to correlate with the vaspin level (r = 0.225, p = 0.076), and a significant relationship was found between chemerin and resistin levels (r = 0.364, p = 0.003) (Fig 3). Finally, when we investigated the correlation of serum adipokine levels with the presence of TNF-α and IL-6, which are representative inflammatory cytokines, differences were only noted in chemerin levels according to the presence or absence of IL-6 (p<0.05) (Fig 4).

## 4. Discussion

A high amount of evidence now indicates that adipokines could be potential biomarkers in various autoimmune disorders. Bustos Rivera-Bahena et al. demonstrated that circulating levels of leptin and resistin, but not those of adiponectin, are independently associated with the clinical activity of RA [22]. Furthermore, it has also been reported that chemerin levels are correlated with disease activity in RA [23]. Likewise, Dini et al. demonstrated that adiponectin is elevated in patients with SLE, even though the correlation of adiponectin levels with disease activity was not shown [24]. Additionally, resistin levels are associated with inflammation and renal disease in SLE, even though a clear difference was not noted between patients and controls [25]. Moreover, a study by Lourenço et al. revealed that leptin directly promotes SLE by increasing antibody production and immunity dysregulation [26], and Elaine et al. demonstrated that chemerin contributes to the pathogenesis of lupus nephritis via dendritic cell recruitment [27]. Nonetheless, given the inconsistent results for a single disease and discrepancies across diseases, more research is needed on the link between adipokines and autoimmune diseases. In the present study, the clinical association between four adipokines in patient sera and the clinical and laboratory features of MPA and GPA were investigated. We found that among the adipokines, serum adiponectin, chemerin, and resistin level was significantly higher in patients with MPA and GPA than in those of HCs. We also identified differences in the expression levels of adipokines in the sera of the patients with MPA and GPA and suggested that the serum resistin level may serve as a biomarker of disease activity.

Similar to cytokines, which possess pro-inflammatory and anti-inflammatory properties, it is now increasingly accepted that adipokines are classified according to their functional effects on the immune system [28]. For example, adiponectin and vaspin are adipokines that play a protective role in inflammation, whereas chemerin and resistin promote the inflammatory process. Mechanistically, adiponectin can act as an inhibitor of the inflammatory response via multiple pathways [29], whereas vaspin is implicated in restoring endothelial progenitor cell dysfunction [30]. On the contrary, resistin induces the production of inflammatory cytokines and affects endothelial cell growth and migration [31], while chemerin functions as a chemoattractant for monocytes and dendritic cells, thereby promoting inflammation [32]. Our results revealed a significant correlation between chemerin and resistin levels, and a tendency of a positive correlation between adiponectin and vaspin, even though a statistical significance was not found. Notably, we found that serum resistin levels were significantly associated with the disease activity measures BVAS and FFS, as well as WBC count and acute phase reactants ESR and CRP, suggesting that these levels could be a surrogate marker reflecting disease activity in MPA and GPA. Conversely, a previous study by Kümpers et al. demonstrated that the pro-inflammatory adipokine leptin is decreased and the anti-inflammatory adipokine ghrelin is increased in patients with active AAV [33]. The reason behind this dissimilarity is unknown; however, it could possibly be related to the bi-directional immunological effect of adipokines, which cannot be defined in a uniform manner [21, 34]. Besides, as it is still not well established how adipokines are produced and how they exhibit a role in MPA and GPA, it seems apparent that the role of adipokines will be addressed better through future studies.

Aging and alterations in metabolism is an important process that affects the function of adipose tissue and the synthesis of adipokines [35]. Given that MPA and GPA usually occur in elderly patients, we compared the serum levels of adipokines among patients of various ages. However, there was no significant difference, suggesting that the influence of age on the serum adipokine levels in our study population might not have been substantial. Next, we also investigated the association between adipokine levels and glucose, lipid profiles, and BMI; however, no association was found except for the levels of adiponectin and HDL cholesterol, which is a lipoprotein that has potent anti-inflammatory effects similar to those of adiponectin [36]. In addition, on performing a partial correlation analysis using BMI ─ which is generally regarded as an index indicating obesity ─ as a covariate, we found that the association between resistin and BVAS was lost; meanwhile, as shown in a previous study, when evaluating the relationship between disease activity and adipokines taking into consideration of BMI, it was revealed that BMI-adjusted resistin (resistin/BMI) was still associated with BVAS, indicating that a complex relationship is present concerning adipokines, adiposity, and disease activity in MPA and GPA.

In the correlation analysis, we found that serum creatinine was correlated with adiponectin, chemerin, and resistin. To our surprise, besides the levels of chemerin and resistin, serum adiponectin was found to be positively correlated with serum creatinine levels. Since serum creatinine levels generally rise in cases with renal involvement, which is the most life-threatening organ manifestation in MPA and GPA, this result was entirely unexpected. However, it was shown in a previous study that adiponectin is related to kidney involvement in autoimmune diseases, as adiponectin levels were found to be elevated in the sera and urine of SLE patients [37, 38]. Alternatively, a positive correlation between adiponectin and creatinine could be present because adiponectin levels increase following the decrease in renal function in patients, including those undergoing dialysis [39, 40]. Moreover, even though chemerin and resistin could be associated with renal function by reflecting a higher degree of inflammation in MPA and GPA, other adipokines could also be affected by impaired renal function; therefore, whether kidney function directly influences in the levels of adipokines should also be investigated [41].

TNF-α and IL-6 are representative pro-inflammatory cytokines implicated in the pathogenesis of chronic autoinflammatory diseases [42], and they are both suggested to play an essential role in AAV. TNF-α expression is increased in the leukocytes and kidney tissues in AAV, which may promote autoantigens of MPO and PR3. In addition, TNF-α is responsible for the adhesion of leukocytes on the endothelium via increased expression of endothelial adhesion molecules [43]. In contrast, IL-6 is a multifunctional cytokine that is capable of regulating the immune response by affecting the production of acute-phase reactants and immunoglobulins [44]. However, in our analysis, we found that the majority of our patients did not have TNF-α in their serum and their IL-6 serum level was not high (median 1.5 pg/mL [IQR 0.0–24.7]), suggesting that the expression of these cytokines in the sera may not be clinically relevant to disease activity. Indeed, the correlation between the absolute titers of TNF-α and IL-6 with BVAS was very low (r = -0.130, p = 0.310 and r = -0.089, p = 0.488). Moreover, when we compared the serum level of adiponectin, resistin, and vaspin between patients positive and negative for TNF-α and IL-6, no significant difference was found. A difference was only evident in chemerin levels between patients positive and negative for IL-6, suggesting a complicated temporal relationship between adipokines, TNF-α, and IL-6.

There are several limitations to this study. First, this was an exploratory study that included a relatively small number of patients which could have influenced the interpretation of the study results. Second, even though we used glucose, lipid profiles, weight, and BMI to assess the metabolic status of our patients, we could not provide detailed data of adiposity such as fat percentage and fat mass. Third, given that three-fourths of our patients were on concomitant medications to treat MPA and GPA, it could have affected in the circulating levels of adipokines. Fourth, the causal relationship between adipokines and inflammation in MPA and GPA could not be elucidated in our study. Additional studies are warranted to elucidate the relationship between adipokines and the pathogeneses of MPA and GPA.

## 5. Conclusions

In conclusion, we demonstrated here, that the levels of adipokines in the sera of patients with MPA and GPA differ depending on clinical and laboratory features, and serum resistin is a potential biomarker reflecting disease activity in MPA and GPA.

## Supporting information

S1 File Original data that was used in the study. (XLSX)

S1 Fig Estimation of the optimal cut-off value of resistin level for predicting high BVAS and FFS using receiver-operating-characteristic analysis. The optimal cut-off value of the resistin level for identifying high BVAS (A) and FFS (B) was estimated. BVAS: Birmingham vasculitis activity score, FFS: Five factor score, AUC: Area under the curve, CI: Confidence interval.
(TIF)

## Abbreviations

AAV: Anti-neutrophil cytoplasmic antibody-associated vasculitis
ANCA: Anti-neutrophil cytoplasmic antibody
BMI: Body mass index
BVAS: Birmingham vasculitis activity score
CRP: C-reactive protein
EGPA: Eosinophilic granulomatosis with polyangiitis
ELISA: Enzyme-linked immunosorbent assay
ESR: Erythrocyte sedimentation rate
FFS: Five-factor score
GPA: Granulomatosis with polyangiitis
HC: Healthy control
HDL: High-density lipoprotein
IL: Interleukin
IQR: Interquartile range
LDL: Low-density lipoprotein
MPA: Microscopic polyangiitis
RA: Rheumatoid arthritis
SHAVE: Severance Hospital ANCA-associated VasculitidEs
SLE: Systemic lupus erythematosus
TNF: Tumor necrosis factor
WBC: White blood cell
