# Supplementary figures and images for: Serum adipokine profiles in patients with microscopic polyangiitis and granulomatosis with polyangiitis: An exploratory analysis
Source: PLoS One. 2021 Jul 9;16(7):e0254226. doi: 10.1371/journal.pone.0254226 (PMC8270208; doi:10.1371/journal.pone.0254226)

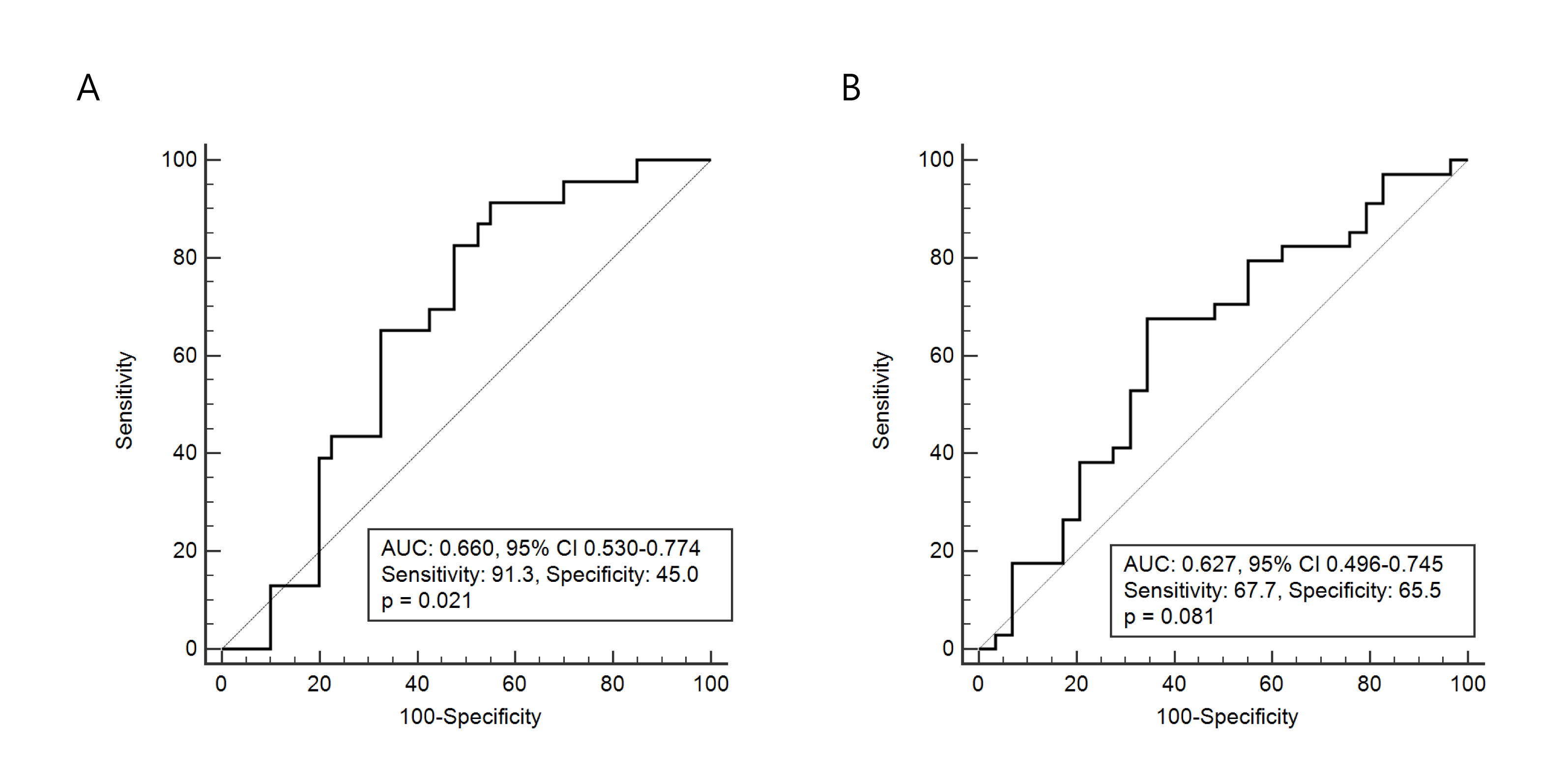

Supplement: S1 Fig — The optimal cut-off value of the resistin level for identifying high BVAS (A) and FFS (B) was estimated. BVAS: Birmingham vasculitis activity score, FFS: Five factor score, AUC: Area under the curve, CI: Confidence interval. (TIF) [file pone.0254226.s002.tif]
